# Peroral Estradiol Is Sufficient to Induce Carcinogen-Induced Mammary Tumorigenesis in Ovariectomized Rats without Progesterone

**DOI:** 10.1371/journal.pone.0162662

**Published:** 2016-09-09

**Authors:** Hillary Stires, Mariana Saboya, Samantha P. Globerman, Wendie S. Cohick

**Affiliations:** Department of Animal Science, Rutgers, The State University of New Jersey, New Brunswick, NJ, United States of America; University of Tennessee Health Science Center, UNITED STATES

## Abstract

A role for estrogens in breast cancer is widely accepted, however, recent evidence highlights that timing and exposure levels are important in determining whether they elicit harmful versus beneficial effects. The rat chemical carcinogen model has been widely used to study the effects of estrogens but conclusions on the levels that lead to tumor development and an absolute requirement for progesterone (P4) are lacking. A newer method of hormone administration mixes hormones with nut butter for peroral consumption allowing for a less stressful method of long-term administration with lower spikes in serum estradiol (E2) levels. The present study was designed to determine if estrogens alone at a physiological dose can drive carcinogen-induced tumors in ovariectomized (OVX) rats or if P4 is also required using this method of hormone administration. Short-term studies were conducted to determine the dose of estrogen (E) that would lead to increased uterine weight following OVX. Subsequently, rats were OVX on postnatal day (PND) 40 then treated daily with E (600 μg/kg/day), P4 (15 mg/kg/day), or the combination. On PND 50, all rats were injected with nitrosomethylurea to induce mammary tumors. Uterine weights, body weights, and serum E2 levels were measured to demonstrate the efficacy of the method for increasing E2 levels during long-term treatment. After 26 weeks, tumor incidence was similar in Sham, E, and E + P4 animals indicating that E was sufficient to induce tumorigenesis when hormone levels were normalized by this method. This study demonstrates peroral administration can be used in long-term studies to elucidate relationships between different types and levels of steroid hormones.

## Introduction

Elucidating the role of female sex steroids in carcinogenesis is critical for general preventive health care in women as well as for understanding the basic pathophysiology of breast cancer. It is widely-accepted that increased exposure to estrogen over a woman’s lifespan increases her risk of breast cancer [1]. A specific role for estrogens in the development of luminal breast cancer is demonstrated by the finding that treatment of intact rats with tamoxifen or aromatase inhibitors (AIs) before nitrosomethylurea (NMU) or 7,12-dimethylbenz(a)anthracene (DMBA) administration attenuates carcinogen-induced tumor development [2, 3]. However, how moderate fluctuations in circulating estrogen levels within a normal physiological range influence cancer incidence is not well understood. A variety of methods are used to administer steroids in ovariectomized (OVX) rodents including silastic tubing, hormone pellets, and daily subcutaneous injections. These methods initially result in supraphysiological levels of hormone [4] which may be a confounding factor since short-term exposure to high levels of estrogens can prevent tumor development [5]. Introducing hormones perorally in nut butter is a newer method of hormone delivery that is less stressful than oral gavage or injections and leads to more normalized levels of ovarian hormones following ovariectomy, without an initial supraphysiological spike in blood concentrations [4]. This method also has the advantage that it mimics oral administration of hormone replacement therapy (HRT), the most common method of delivery in women. Oral administration requires higher doses of steroids to elevate systemic levels due to extensive metabolism by the gut and liver, which can also result in a different metabolic profile from that of other routes of administration [6, 7]. However, to our knowledge, peroral administration has not yet been used in rodent studies to study the role of estrogen in carcinogen-induced tumor development.

Similar to estrogens, progesterone (P4) is secreted from the ovary, cycles over a woman’s lifespan and decreases in menopause. Whether P4 plays a role in carcinogen-induced tumorigenesis has not been studied as extensively as estrogens. The intact ACI rat spontaneously develops tumors in response to high doses of estrogens but high concentrations of both estrogens and P4 are required for the response in OVX animals [8]. The importance of P4 in other studies using OVX rodents is unclear [9, 10]. As with estrogen, divergent results may be due to both the mode of administration and the serum concentrations obtained with different methods.

Given that peroral hormone administration represents a less-stressful method of hormone administration that results in more physiological levels of estrogen and mimics oral HRT, the overall goal of this study was to determine its usefulness for long-term carcinogen studies. A specific goal of the study was to determine if estradiol (E2) alone can restore carcinogen-induced tumorigenesis or if P4 is also required with this method. Short-term studies were conducted to determine the oral dose of E2 that was estrogenic, which was then used for a long-term study. Results indicated that long-term peroral hormone replacement following OVX leads to physiological levels of E2 that are sufficient to restore tumorigenesis in response to NMU without P4.

## Materials and Methods

All animal procedures were approved by the Rutgers University Institutional Animal Care and Use Committee according to NIH guidelines. All surgery was performed under isoflurane gas and all efforts were made to minimize suffering. During surgery, animals were given Bupivacaine (Henry Schein; Dublin, OH) as a local anesthetic. Post-operative care included local administration of Buprenex (Henry Schein) as a local analgesic followed by daily monitoring for two weeks. For tumor development studies, animals were sacrificed if their tumor volume (as measured by S^2^ * L/2 where S and L are the shortest and longest diameter in mm, respectively) exceeded 10% of body weight or the mean tumor diameter exceeded 40 mm.

### Preparation and Administration of Hormones

E2, E2 benzoate (EB), and P4 (Sigma-Aldrich; St. Louis, MO) were independently dissolved in ethanol prior to dissolution in sesame oil (Sigma-Aldrich) at a concentration of 600 μg/ml (E2 or EB) and 15 mg/ml (P4). Equivalent volumes of ethanol without hormone were added to sesame oil to serve as the vehicle control for each hormone. Body weights were obtained prior to feeding each day and used to determine the appropriate volume of hormone to administer to each animal. Each day the appropriate volume of hormone or vehicle was mixed into approximately 4 g of peanut butter (Skippy Natural Peanut Butter) placed on 2x2 inch squares of parchment paper. Each animal was offered two allotments of peanut butter to achieve the appropriate treatment combination e.g. E2 and vehicle, P4 and vehicle, E2 and P4, or vehicle and vehicle. The animals had access to the treatment until the next day when old papers were removed and new treatments were given. Hormone consumption was evaluated each morning before administering new treatments based on visualization of whether the paper was completely devoid of peanut butter (all), peanut butter was partially consumed (partial), or appeared the same as it had when hormones were introduced the previous day (none).

### Pilot Studies to Determine Dose of EB

Female Sprague Dawley rats (Charles River; Wilmington, MA) arrived on post-natal day (PND) 28 and were housed in a controlled environment with *ad libitum* access to food (Purina Mills Lab Diet; St Louis, MO) and water. To acclimate animals to the peanut butter, rats were offered peanut butter without treatment 2 hours after lights on each morning beginning on PND 35. Groups were normalized by body weight prior to surgeries, which were performed on PND 40 or 41. Bilateral ovariectomy (OVX) or sham surgery was carried out under isoflurane gas. Surgical procedures were performed using the aseptic no-touch technique. Hormone treatments were started the day after surgery. Since the half-life of E2 in serum is only 2 to 8 hours [11, 12], EB was chosen due to its longer half-life. For the first pilot study, OVX rats were divided into three treatment groups (n = 4): 1) EB (150 μg/kg/day); 2) EB + P4 (15 mg/kg/day); and 3) vehicle. A fourth group was given vehicle following sham surgery (n = 4). In the second pilot study, OVX rats were divided into 3 treatment groups: 1) EB (300 μg/kg/day; n = 5); 2) EB (600 μg/kg/day; n = 5); and 3) vehicle (n = 3) with a fourth group that received vehicle following sham surgery (n = 3). After 10 days of treatment, rats were sacrificed by rapid decapitation. The uteri were excised and defatted then weighed. For sham animals, ovaries were removed from the uterine horns prior to weighing.

### NMU Tumor Study

Rats arrived from Charles River on PND 28 and were housed and maintained as described above. Groups were normalized by body weight on the day of surgery performed on PND 40 or 41. OVX rats were subsequently treated with EB (600 μg/kg/day; n = 8), P4 (15 mg/kg/day; n = 7); EB + P4 (n = 8); or vehicle alone (OVX; n = 8) starting the day after surgery. A 5th group was given vehicle following sham surgery (Sham; n = 8). On PND 50, rats were injected I.P. with 50 mg/kg NMU (Sigma-Aldrich). All injections were completed within one hour of dissolving NMU in sterile 0.9% saline (pH 4). Rats were palpated for tumors biweekly starting 4 weeks post-NMU injection and measured using a Vernier caliper. Tumor volume was measured and calculated as described above. Body weights were recorded biweekly. Rat chow was kept in hoppers for the duration of the study to monitor food consumption biweekly. Blood was taken from the lateral tail vein 4, 8, and 12 weeks after NMU injection 3 to 5 hours after hormones were offered. Several animals were sacrificed early because their tumor volumes exceeded 10% of their body weight. This included two rats from the group treated with EB/E2 (E) (sacrificed 13 and 25 weeks post NMU), one rat from the E + P4 group (sacrificed 24 weeks post NMU) and one rat from the Sham group (sacrificed 16 weeks post NMU). Remaining rats were sacrificed by rapid decapitation twenty-six weeks after NMU injection. Tumors were excised and stored in 10% neutral buffered formalin (NBF). Pelts were removed then stretched, pinned to a wooden board, and submerged in 10% NBF. Uteri were excised, fat was removed, and uteri were weighed. For Sham animals, ovaries were removed from the uterine horns prior to weighing. Images of the uteri were taken prior to storage in 10% NBF.

### Serum E2 Levels

Blood taken from the lateral tail vein was allowed to clot for 30 minutes at room temperature then spun at 1500 x g for 10 minutes at 4°C. Serum was collected and stored at -80°C until further analysis. E2 levels were determined using an ELISA following the manufacturer’s directions (Calbiotech; Spring Valley, CA). All samples from the E only and E + P4 groups were analyzed, while a subset of Sham animals were included for comparison. For OVX animals, sera from individual animals were combined to generate four pools for analysis.

### Mammary Gland Whole Mount Analysis

After fixing pelts in 10% NBF for at least 10 days, the left fourth inguinal mammary gland was dissected away from the skin, stretched on slides, and allowed to air-dry for 30 min. If this gland contained a tumor, the contralateral gland was used for analysis. Glands were then rehydrated in 70%, 50%, and 25% ethanol, placed in H_2_O for 5 min, and stained in carmine alum for 1–2 days (Sigma-Aldrich). After staining, slides were dehydrated in 70% and 95% ethanol followed by xylene. Glands were cleared in toluene for 1–6 weeks after dehydration to remove excess fat then air dried for 30 minutes before mounting in SealPAK pouches (Kapak; Minneapolis, MN) with cedar wood oil (Acros; Geel, Belgium). Whole mounts were imaged using a Nikon DS-Fi1 camera (Nikon; Melville, NY) with NIS Elements software (Nikon). The length of the mammary parenchyma was measured using the straight line function in FIJI Is Just ImageJ (FIJI) [13] from the far edge of the lymph node to the most distal point of the ductal structure of the gland. To enhance magnification, glands were viewed and images taken using a Leica MDG41 stereomicroscope (Leica; Buffalo Grove, IL).

### Tissue Histology

Fixed tumor and uterine tissues were dehydrated, cleared, and embedded in Paraplast using facilities located in the Histopathology Core of the Environmental Occupational Health Sciences Institute at Rutgers University. Samples were sectioned at 6 μm and placed on slides. For uteri, cross sections were obtained. Slides were baked for 15 minutes at 60°C, followed by deparafinization in xylene and rehydration in decreasing concentrations of ethanol. Slides were stained with hematoxylin and eosin then mounted with Permount. A toxicological pathologist, who was blind to treatment, viewed tumor slides to determine whether the tumors were adenomas or adenocarcinomas based on the most malignant portion of each tumor following previously defined criteria [14]. Uteri were scored to quantitate the grade (0–3) and stage (0–3) of metaplasia, which were added together to obtain an overall score. Representative images of 0+0, 1+1, 2+2, and 3+3 were taken using an Olympus FSX100 microscope (Olympus; Waltham, MA).

### Immunohistochemistry (IHC)

Fixed tumor tissue was dehydrated, cleared, and embedded as described above. Slides were baked at 55°C for 30 minutes, deparaffinized in xylene, and rehydrated in decreasing concentrations of ethanol. Antigen retrieval was performed by boiling slides in 0.01 M sodium citrate buffer for 30 minutes then cooled at room temperature to 45°C before proceeding.

For Ki67, tissues were blocked in normal donkey serum (Santa Cruz Biotechnology; Dallas, TX) for 30 minutes at room temperature. Samples were incubated overnight at 4°C with either rabbit-Ki67 primary antibody (1:100, ab16667; Abcam; Cambridge, MA) or rabbit primary antibody isotype control (Life Technologies; Grand Island, NY), which served as a negative control on each slide. The following day the tissues were incubated with Alexafluor 488 labeled donkey anti-rabbit secondary antibody (Life Technologies) for 60 minutes at room temperature. Slides were counterstained with DAPI (Life Technologies) then mounted with Prolong Gold Antifade (Life Technologies).

For estrogen receptor α (ERα) and P4 receptor (PR), endogenous peroxidase activity was blocked using 3% H_2_O_2_ for 10 minutes. ERα sections were blocked with normal goat serum (Vector Laboratories; Burlingame, CA) following the manufacturer’s directions while PR was blocked with normal horse serum (Vector Laboratories) in a 1:1 dilution. Rabbit ERα primary antibody (MC20; Santa Cruz Biotechnology) was diluted in 1% BSA to 1:500 while Mouse PR primary antibody (MS-197-P0; ThermoScientific) was diluted in PBS + 0.1% triton to 1:300. Rabbit and mouse primary antibody isotype control served as negative controls for ERα and PR, respectively. Secondary antibodies were applied according to manufacturer’s directions followed by development in 3,3'-diaminobenzidine (Sigma-Aldrich). Slides were counterstained with hematoxylin, dehydrated with increasing concentrations of ethanol followed by xylenes, then mounted using Permount.

For all IHC, tumor sections were viewed and five representative pictures were taken at random from one section per tumor using an Olympus FSX100 microscope at 20X magnification. Ki67 pictures were taken with the same exposure settings for all samples using green and blue fluorescence channels to visualize antibody staining and nuclei location, respectively. For ERα and PR, pictures were taken using bright field setting.

The amount of Ki67 staining per nuclei was calculated using previously described methods [15]. ERα and PR were analyzed using the color deconvolution plug-in of [13]. Briefly, images were split into brown and purple images using the H DAB vector. Mean grey density was determined from the brown image then converted to optical density using the equation OD = log (255/mean grey density), where 255 is the maximum intensity for 8-bit images. OD values from the 5 separate images per tumor were averaged to give a tumor OD. For animals with multiple tumors, Ki67 staining or ERα and PR OD values for all of their tumors were averaged.

### Statistical Analysis

Body weights were analyzed using a repeated measures ANOVA followed by a post hoc Bonferroni-Dunn multiple comparison tests through 13 weeks when the first rat was sacrificed. Body weights were also analyzed on the final day of the study by one-way ANOVA with Newman-Keuls post hoc analysis. Uterine weights, tumor burden, and mammary gland length were analyzed using a one-way ANOVA with Newman-Keuls post hoc analysis. Tumor incidence was analyzed using a Mantel-Cox Log-rank test. Tumor type was analyzed using a Chi-square analysis. Uterine score, Ki67 expression, ERα expression, and PR expression were analyzed using a Kruskal-Wallis test with Dunn’s Multiple Comparisons posttest or a Mann Whitney test. GraphPad Prism version 5.0 (La Jolla, CA) was used to perform statistical analyses and p ≤ 0.05 was considered significant.

## Results

### Short-term Studies

**Fig 1:**
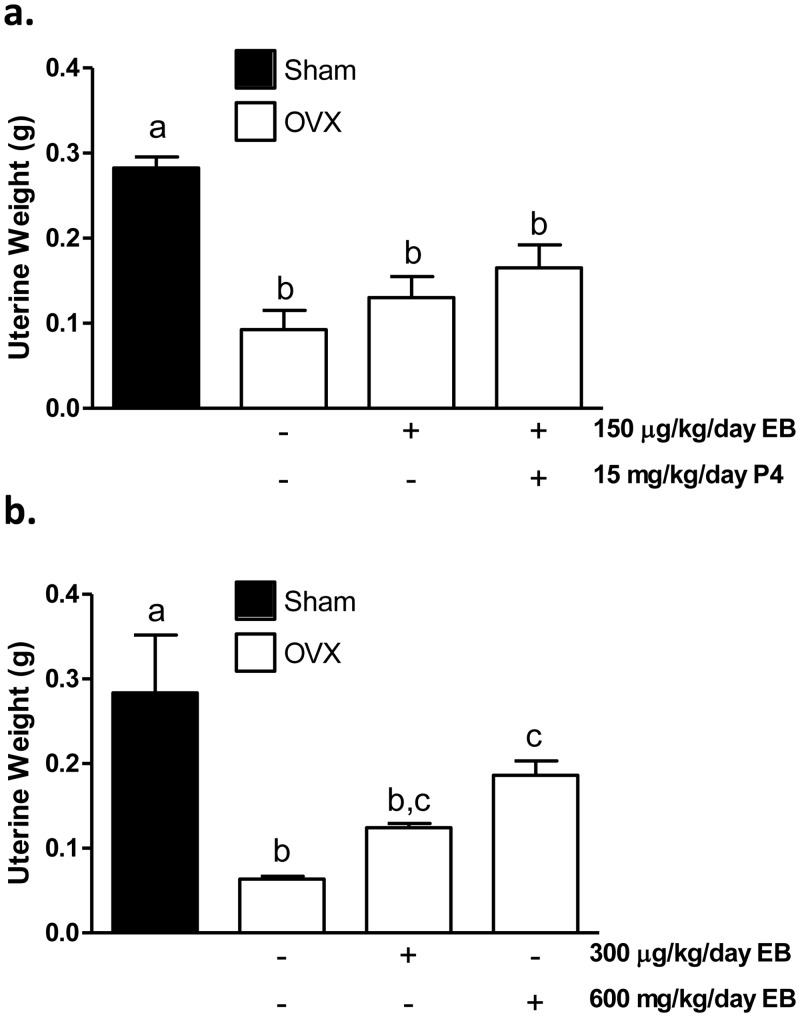
The most effective dose of EB for peroral administration following OVX is 600 μg/kg/day. Uteri were excised and uterine wet weights were determined at sacrifice (for Sham animals, ovaries were removed prior to weighing). Uterine weights are expressed as mean ± SEM, p < 0.05, one-way ANOVA; Newman-Keul’s posttest; different letters denote significant difference. (a) In the first short-term study, uterine weights of hormone-treated rats were not different from OVX (n = 4). (b) In the second study, 600 μg/kg/day EB increased uterine weight over OVX (n = 5, 3, and 3 for EB treated, Sham, and OVX animals respectively).

To determine the dose of peroral EB that would restore E2 to physiological levels in OVX animals, two short-term studies were conducted using uterine weights as a measure of estrogenicity. As expected, uterine weights in the OVX group were less than those in the sham group ten days after surgery in both studies. In the first study, 150 μg/kg/day EB alone or in combination with 15 mg/kg/day P4 was unable to restore uterine weights over OVX (Fig 1a). In the second study, daily treatment with 300 μg/kg EB perorally was unable to rescue the decrease in uterine weight caused by OVX, while the 600 μg/kg dose increased uterine weight over OVX (p < 0.05; Fig 1b). Therefore, the 600 μg/kg daily dose was chosen for the long-term tumor study.

### Long-term NMU Study

#### Hormone Consumption

Rats were OVX on PND 40 and treated daily with hormones until 26 weeks post NMU injection. Hormone consumption over the first five weeks of the study revealed that animals treated with EB (either alone or in combination with P4) consumed “partial” or “none” of both of their treatments while animals treated with vehicle alone or with P4 licked their papers clean each day. The observation that animals consumed the entire vehicle or P4 treatments suggested that the animals had a taste aversion to either the E2 or benzoate component [16] of the EB. Therefore, animals treated with EB were switched to E2 starting 4 weeks post NMU. This change did not seem to correct the problem as animals treated with E2 + vehicle or E2 + P4 continued to consume partial hormone for the remainder of the study.

#### Uterine and Body Weights

**Fig 2:**
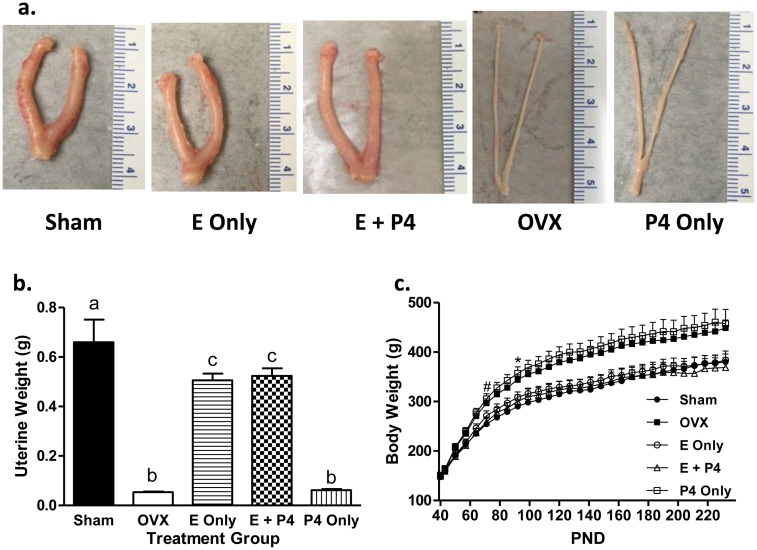
Long-term daily peroral E treatment is physiologically active. At sacrifice, uteri were excised and weighed. Images of uteri were taken before storage in 10% NBF. (a) Representative images of uteri from each treatment group. (b) Uterine weights were reduced in OVX rats treated with vehicle but increased with E or E + P4 treatment. P4 alone was unable to restore uterine weights. Uterine weights are expressed as mean ± SEM; n = 8, 7 for P4 only; p < 0.05, one-way ANOVA; Newman-Keul’s posttest; different letters denote significant difference. (c) Rats were weighed biweekly for the duration of the study. Long-term exposure to E or E+ P4 resulted in body weights that were similar to those of Sham controls. Daily treatment with P4 did not prevent weight gain following OVX. Body weights are expressed as mean ± SEM, n = 8, 7 for P4 only; repeated measures ANOVA through the first animal sacrifice at 13 weeks post NMU (PND 148) (treatment: p < 0.01) with Bonferroni-Dunn multiple comparisons test (* start of p < 0.05 for OVX, # start of p < 0.05 for P4 only; compared to Sham). The pattern persisted in the remaining animals at the end of the study (p < 0.05, one-way ANOVA, Newman-Keul’s posttest).

Uteri from Sham animals and animals treated with E or E + P4 appeared normal and were similar upon visual inspection. Uteri from OVX animals treated with or without P4 were atrophied and visually indistinguishable from each other (Fig 2a). As expected, uterine weights were significantly decreased in OVX compared to Sham animals (Fig 2b). Treatment with E or E + P4 increased uterine weights over OVX while treatment with P4 alone did not affect uterine weight (p < 0.05; Fig 2b). Body weights of OVX and P4 only groups were different from those of Sham beginning at 4 and 7 weeks post NMU injection, respectively (p < 0.01), while those of E only and E + P4 were not different from Sham controls through 13 weeks post-NMU injection when the first animal (of four total) needed to be sacrificed early due to large tumor size (p < 0.05; Fig 2c).

#### Serum E2

**Fig 3:**
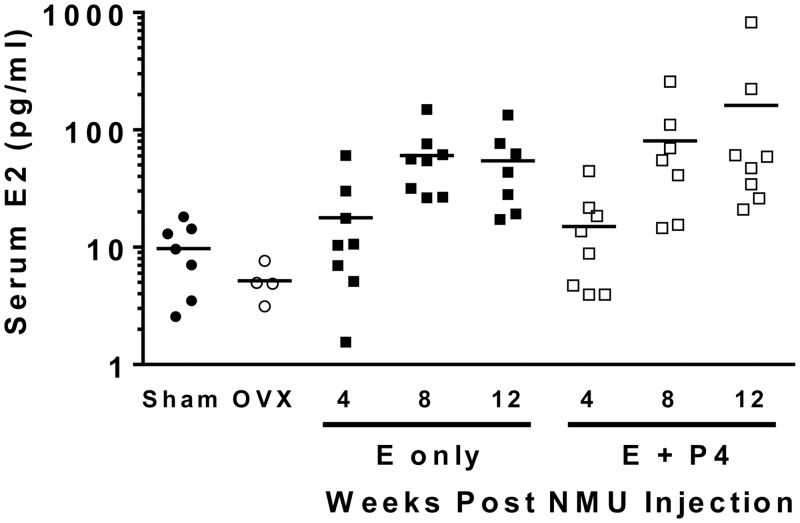
Serum E2 levels are elevated with peroral hormone treatment. Blood samples were drawn from the lateral tail vein 5, 3, and 5 hours after treatments were presented 4, 8, and 12 weeks after NMU injection, respectively. Blood draws from all animals were completed within 2 hours on each of the three days. Serum was analyzed using an E2 ELISA (Calbiotech). Blood from one animal in the 12-week E only group and one animal in the 8-week E + P4 group hemolyzed, thus these samples were not included in the analysis. All values from E and E + P4 animals are presented to demonstrate the range of serum E2 across animals and treatments while a subset of Sham animals and serum pools from OVX animals were included for comparison. Horizontal lines indicate mean values for each time point.

To monitor serum E2 levels, blood was collected from rats via the lateral tail vein from all animals beginning either 3 or 5 hours after hormones were administered at 4, 8 and 12 weeks post-NMU injection. Serum from E only and E + P4 groups was analyzed at all time points while a subset of samples from Sham animals and serum pools from OVX animals were assayed for comparison. The interquartile range of E2 levels of Sham and OVX were 3.5 to 14.2 pg/ml and 3.6 to 7.0 pg/ml, respectively. The interquartile range of serum E2 levels for the E only group was 17.2 to 61.4 pg/ml and 14.5 to 60.8 pg/ml for the E + P4 group (Fig 3).

#### Uterine Histology

**Fig 4:**
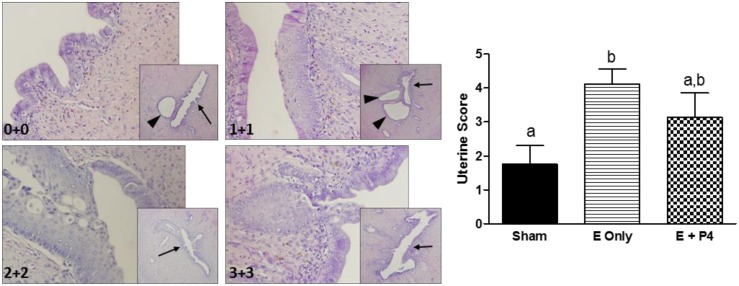
Treatment with E leads to increased uterine metaplasia in treated animals. Cross sections of uteri were stained with hematoxylin and eosin. A toxicological pathologist staged and graded the slides from 0–3 for each category leading to scores of stage + grade, specifically focusing on the uterine lumen (arrows) and not on dilated ducts (arrow heads). Images are representative of 0+0 (relatively normal), 1+1, 2+2, and 3+3 respectively. The larger images were taken at 20X magnification and the inset at 4X magnification. Scores were derived by adding stage and grade to get a final score of 0–6. P4 only and OVX animals were not included in analysis as all uteri were atrophied and not metaplastic. Uterine scores are expressed as mean ± SEM; n = 8; p < 0.05, Kruskal-Wallis test with Dunn’s Multiple Comparison’s posttest; different letters denote significant difference.

Since serum E2 values of animals treated with E were elevated over values of Sham controls, uterine morphology was analyzed to determine if there were any abnormalities induced by E2 treatment. P4 only and OVX animals were not included in the analysis since their uteri were atrophied and did not display any metaplasia. Visual observation of uterine sections from E only, E + P4, and Sham animals revealed epithelial metaplasia that was extensive in some samples and less apparent in others. To quantify the changes, a toxicological pathologist determined how extensive the injury was (stage) and how disorganized the cells were (grade). Representative images are presented in Fig 4. Stage and grade were added together to give an overall uterine score and scores were compiled by group. There was more extensive uterine metaplasia in the E only group compared to Sham (p < 0.05). The metaplasia in the E + P4 group tended to be less extensive than in the E only group, but still more extensive than in Sham group.

#### Mammary Glands

**Fig 5:**
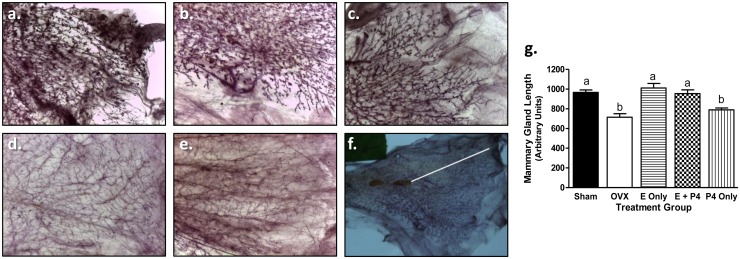
Mammary gland development of E and E + P4 treated animals is similar to Sham controls. Representative images of whole mounts (4X magnification) demonstrated ductal thickness was similar in Sham (a), E only (b), and E + P4 (c) while the overall morphology of glands was similar between OVX (d) and P4 only (e) with thinner ducts relative to the other groups. The length of the mammary gland parenchyma was measured from the lymph node to the most distal edge of the ducts using FIJI as shown in (f). The parenchyma from Sham, E only, and E + P4 glands exhibited similar ductal lengths while OVX and P4 only were shorter (g). Mammary gland length is expressed as mean ± SEM; p < 0.05, one-way ANOVA; Newman-Keul’s posttest.

Since E2 and P4 are important for normal mammary gland growth and maintenance, mammary gland whole mounts were prepared and imaged to analyze morphology. As expected, the ductal structures of the glands in the Sham group extended to the end of the fat pads with branching and alveolar buds appropriate for adult virgin animals (Fig 5a). The mammary glands of animals treated with E or E + P4 appeared similar (Fig 5b and 5c). The glands from the OVX and P4 groups were indistinguishable from one another and exhibited very thin ducts and lacked alveolar buds (Fig 5d and 5e). Parenchyma of mammary glands from OVX ± P4 animals did not extend as far into the fat pad relative to the parenchyma of the Sham and E or E + P4 glands (Fig 5f and 5g).

#### Tumor Development

**Fig 6:**
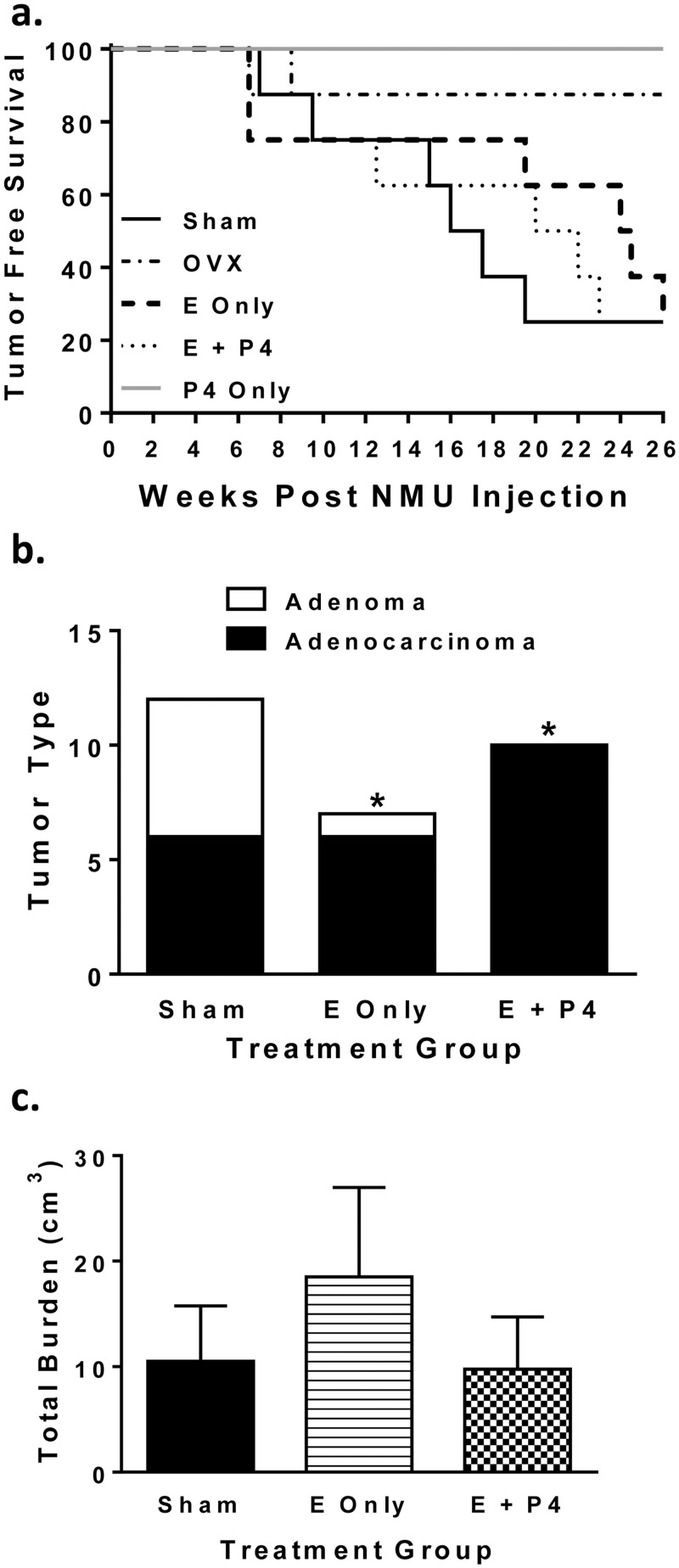
Peroral treatment with E and E + P4 after OVX results in similar tumor incidence but more adenocarcinomas following NMU relative to Sham. (a) Rats were OVX on PND 40, injected with NMU on PND 50, and palpated biweekly for the appearance of tumors. There was no difference in tumor incidence between E, E + P4 and Sham but all three groups were different from P only and OVX, which were not different from one another (p < 0.05, Mantel-Cox Log-rank test). (b) Tumor type was classified by a histological pathologist as adenoma or adenocarcinoma based on the most malignant part of each section. The effect of treatment on tumor type was analyzed using a Chi-square analysis (* p < 0.05). (c) Tumors were measured with a Vernier caliper the morning of sacrifice. To calculate tumor burden, the volume of each tumor was added together per animal. There were no differences in tumor burden among treatment groups.

To determine the influence of long-term peroral hormone treatment on mammary tumor development, rats were injected with NMU on PND 50. Tumors were palpated biweekly and tumor incidence was calculated (Fig 6a). The first tumors appeared 6.5 weeks post NMU injection in E only, E + P4, and Sham groups. There was no difference between tumor incidence in E only, E + P4, or Sham groups 26 weeks post NMU injection. There tended to be an increase in tumor latency in the E only group. One tumor developed in the OVX group 8.5 weeks post NMU injection. All tumors were adenomas or adenocarcinomas. Interestingly, there was a significant difference in tumor type by treatment group with E and E+ P4 treated groups developing more malignant tumors (p < 0.05; Fig 6b). Autopsy revealed that the single OVX animal that presented with a tumor was the only animal that had large metastases to the lungs which was confirmed by histological examination. Total tumor burden was calculated by adding the volumes of individual tumors for each animal. There was no difference in total tumor burden although the E only group tended to have a greater burden than the E + P4 group or the Sham group (Fig 6c).

#### Tumor IHC

**Fig 7:**
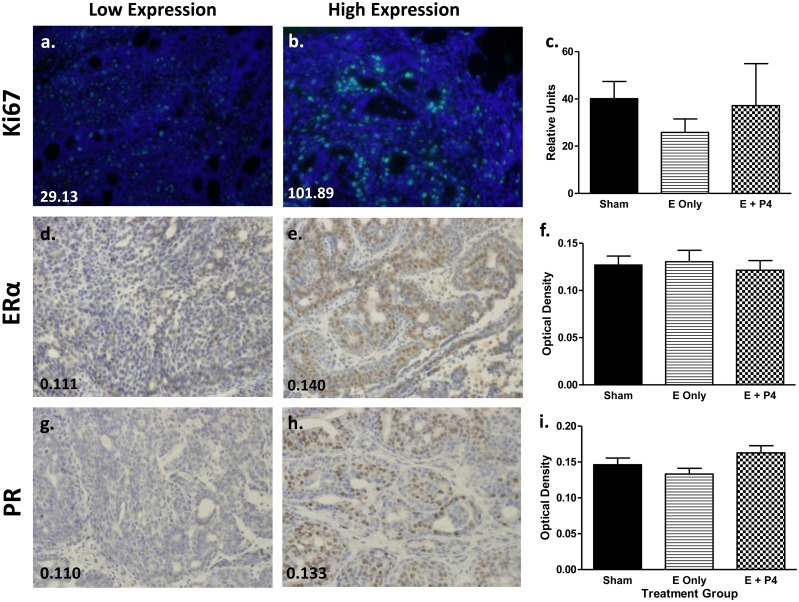
Treatment of OVX animals with E or E + P4 results in tumors with similar proliferation indices and receptor status relative to tumors from Sham controls. Ki67, ERα, and PR IHC was performed and measured as described. Images depicting low (a, d, g) and high (b, e, h) expression of Ki67, ERα, and PR are presented with corresponding scores in the bottom left corner of each image. There were no differences in Ki67 or ERα expression between treatment groups. Animals treated with E + P4 had more PR expression than animals treated with E alone (Mann-Whitney test comparing E only to E + P4, p < 0.05).

Ki67 was analyzed as a marker of proliferation and ERα and PR were assessed to determine hormone receptor status. Representative images of low and high expression levels for each marker are shown in Fig 7. There were no significant differences in Ki67 staining between the Sham, E only, and E + P4 groups, although the E only group tended to be lower (Fig 7c). Similarly, there were no significant differences between groups for expression of ERα (Fig 7f). There was no significant difference in PR expression between the three treatment groups. However, comparing PR expression between E vs. E + P4 indicated expression was greater in animals treated with E + P4 compared to those treated with E only (Mann Whitney test, p < 0.05; Fig 7i). Sham animals had PR expression that fell in the range of the E only and E + P4 groups. The tumor from the OVX animal had one of the highest Ki67 expression levels and the lowest ERα and PR expression (data not shown).

## Discussion

The goals of this study were two-fold: (1) to determine if tumors can be induced using peroral hormone administration as previous carcinogen studies used methods that resulted in supraphysiological levels of hormone or induced stress [4] and (2) to determine if estrogens alone can drive carcinogen-induced tumorigenesis in OVX rats in the absence of P4 using this method of hormone administration. Peroral administration represents a stress-free method to administer hormones that mimics oral hormone ingestion, the most common method of HRT. Oral administration produces a hepatic ‘first-pass’ effect, resulting in extensive metabolism of the hormones as well as stimulation of hepatic factors [6, 7, 17]. These first-pass effects could potentially impact tumorigenesis as they do for cardiovascular and venous function [6, 7, 17]. Therefore peroral administration provides a method of hormone delivery that mimics oral HRT in terms of both the high doses of steroids initially presented to the liver as well as ultimate bioavailability to other tissues. In the current study, body weights and uterine weights following six months of peroral hormone administration indicated that hormones were biologically active over an extended period of time.

Pilot studies were necessary to ensure the dose of E chosen would be sufficiently estrogenic [18]. Previous short-term studies with peroral administration used a lower dose of E2 (28 μg/kg/day) that was unable to restore uterine weights [4]. Generally, higher doses are required for peroral administration since first pass through the liver and gut decreases overall bioavailability [6, 19, 20]. In the present work, only the 600 μg/kg dose increased uterine weights over the OVX animals in a short-term ten-day study and was therefore used for the long-term study.

While peroral feeding of nut butter has been examined as a useful method of hormone administration, this is the first long-term study to examine its effectiveness in a carcinogenic-induced model of tumorigenesis. After a little over a month of feeding, rats treated with EB (E only and E + P4 groups) did not consume all of their treatments and were thus switched to E2 for the remainder of the study with the thought that they had a taste aversion to benzoate, a molecule that has been shown to have a bitter taste [16]. Switching to E2 did not help with consumption, which may have occurred due to an aversion to the taste of E2 or perhaps more likely because the animals had already developed a taste aversion to any treatment in peanut butter. Interestingly, serum E2 levels did not correspond with the pattern of feeding that was observed. It is possible that transdermal exposure from the lipophilic peanut butter infused with hormones increased exposure over peroral consumption alone. In future studies, it would be helpful to do short-term, 4-week studies on taste aversion before beginning a long-term study. However, based on long-term measures of estrogenicity including uterine weights, body weights, and uterine histology, animals treated with E were exposed to sufficient levels of hormone.

To ensure E2 levels were elevated despite a lack of complete consumption, serum E2 levels were measured at three separate time points. The interquartile range of serum levels in E-treated animals ranged from 17.2 to 61.4 pg/ml in the E only group and 14.5 to 60.8 pg/ml in the E + P4 group across the three time points. Previous studies have shown that intact animals sacrificed during estrus or proestrus have serum E2 levels ranging from 2.4 to 145.4 pg/ml [4, 21]. E2 levels in serum of Sham animals in the current study had an interquartile range of 3.5 to 14.2 pg/ml while those of OVX animals ranged from 3.6 to 7.0 pg/ml. The ELISA used in the present work has been reported to be the most accurate and sensitive compared to other commercially available assays for rodent serum [22]. Interestingly, similar to the present results, this assay could not distinguish between Sham and OVX animals based on E2 serum levels in animals that were not specifically determined to be in proestrus [22]. Therefore, while the values reported here for E-treated animals were higher than those of Sham controls in this study, the levels were generally within a physiological range.

Since weekly E2 values represented a single time point for each animal, other endpoints were evaluated to better assess the physiological relevance of the dose used in this study. OVX causes rats to become hyperphagic and increases positive energy balance leading to increased weight gain compared with sham operated animals [23]. Treatment with E2 reduces body weight gain [24–26] while P4 alone has no effect on body weight following OVX [27]. Similar results were found in the present study, indicating long-term efficacy of the E treatment. Uterine weights in the long-term study followed those of the short-term study where OVX decreased uterine weight and treatment with E rescued it. Uterine histology was evaluated as a more sensitive marker of estrogenicity. Uteri were sectioned and stained with H&E to analyze cellular endpoints including luminal epithelial height and gland number [28]. This analysis indicated that uteri did not have a normal histology and exhibited differing levels of metaplasia. While uterine metaplasia in response to NMU has been previously described [29], the metaplastic response was greater in OVX animals treated with E alone relative to Sham controls in the present study. As expected, treatment with E + P4 tended to decrease the metaplasia induced by E alone. These results suggest that a lower dose of E might have been sufficiently estrogenic in a long-term study without inducing uterine metaplasia.

In the present study, mammary gland morphology in OVX animals was maintained with either E or E + P4 treatment. Interestingly, there was not an obvious difference between the two treatments, although again a larger sample size maybe required to detect subtler differences. Mammary gland morphology was not maintained with P4 alone, indicating that there is an absolute requirement for estrogens. Similar results were reported in a recent study that examined E2 ± dydrogesterone (DG) using a similar method of hormone administration. Histological analysis of mammary glands revealed that DG did not reverse the atrophic morphology observed in OVX rats, however, treatment with E2 with or without DG resulted in mammary glands similar to those of sham controls [30]. While the measurement included here was a qualitative overview of mammary gland morphology, these results are interesting as previous research has indicated that E2 is required for pubertal mammary gland development but P4 is also required for mammary gland maintenance during adulthood in mice [31].

A clear role for estrogens is indicated by studies showing that treatment with tamoxifen or AIs leads to tumor regression [3, 32]. However, this experimental paradigm does not determine whether P4 is also required for tumorigenesis nor does it consider the role of the dose or timing of hormone administration. Considerable data supports a proliferative role for P4 in the normal mammary gland [33, 34], however, its role in breast cancer is controversial [34, 35]. The Women’s Health Initiative showed that conjugated equine estrogens (CEE) and medroxyprogesterone acetate (MPA; a synthetic progestin) act synergistically to promote breast cancer compared with CEE alone [36, 37], leading to the hypothesis that P4 increases breast cancer risk. Subsequent studies focusing on the timing of administration and the effects of different types of progestogens [38, 39] indicate that certain progestins may increase breast cancer risk but native P4 may not. These epidemiological studies have increased recognition of the complexities of P4’s role in breast cancer and the need to further elucidate the interactions between estrogen and P4.

Interestingly, few studies have used ovarian ablation and hormone replacement to answer these questions and those that have been performed have shown varying results. In the present study, tumor incidence was similar between Sham, E and E + P groups, indicating that contrary to previous research using a chemical carcinogen in an OVX rat model [9, 10], estrogens alone can fully restore tumorigenesis. Tumors failed to develop in response to NMU when 26–30 mg of E2 was administered to OVX rats using silastic tubing. Tumors did develop in animals treated with estrone and tumor incidence nearly doubled when P4 was also included [10]. The authors attributed the lack of an effect of E2 to the high dosage administered based on reports that high levels of E2 and P4 (similar to levels seen in pregnancy) around the time of NMU administration prevent mammary tumor development in intact rats [40, 41]. The reason for the difference in tumor burden with E2 versus estrone was unresolved. In contrast to findings of Bigsby [10], tumors did form in response to DMBA following ovarian ablation and replacement with daily gavage feeding of 100, 300, or 900 μg/kg/day E2 or daily intravenous administration with 0.4, 10 or 250 μg/kg/day E2; however, total tumor burden was not restored to that of sham animals [42]. The combination of E2 and P4 was not examined in this study and there was no relationship between the dose and tumor incidence [42]. The differences between our study and previous studies might be attributed to the level of circulating hormones achieved by treatment, which were not monitored in previous studies. Alternatively, the timing of hormone administration could influence tumor development. Specifically, silastic tubing provides a constant slow release of hormone while peroral treatment gives a daily pulse of hormone, which better mimics the cyclic fluctuations in steroids. In future studies it would be worthwhile to compare multiple types of hormone administration to better understand hormone kinetics and their effects on long-term physiology.

Overall tumor burden, tumor multiplicity, and tumor phenotype also did not differ between the Sham, E and E + P4 groups. Differences may be masked by the small sample size which was reduced even more by the fact that not all animals in each group developed tumors. While gross tumor parameters did not differ between these groups, E-treated animals tended to have a delay in tumor onset (i.e. latency) indicating that P4 may play a role in tumor initiation or enhancing tumor promotion. Animals treated with E or E + P4 had more adenocarcinomas than the Sham group suggesting the elevated levels of hormone may increase malignant potential of these tumors. Interestingly, tumors from the E + P4 group tended to have increased PR expression compared to the E only group. In the normal mammary gland, treatment with P4 downregulates expression of PR [43]. However, tumors do not always respond to hormones in a similar manner as the normal gland. For example, cells that express ERα and PR are limited and do not typically divide, but act in a paracrine manner to stimulate the division of neighboring cells [44, 45]. Luminal breast tumors have increased levels of ERα and PR and the cells that express these receptors also divide. The switch from paracrine signaling to autocrine signaling is an active area of research.

In conclusion, E treatment alone was found to be sufficient for restoration of carcinogen-induced tumorigenesis when OVX rats were given replacement hormone perorally. Endpoints of E2 efficacy demonstrate the usefulness of this approach in terms of providing a low-stress method of hormone administration that can be used to achieve circulating concentrations of physiological levels of hormones. This model will be useful in future studies designed to understand the effects of combinations of progestogens and estrogens on mammary tumor development.

## References

[1] Hilakivi-Clarke L, de Assis S, Warri A. Exposures to synthetic estrogens at different times during the life, and their effect on breast cancer risk. J Mammary Gland Biol Neoplasia. 2013;18(1):25–42. 10.1007/s10911-013-9274-8 . 23392570 PMC3635108

[2] Lubet RA, Steele VE, DeCoster R, Bowden C, You M, Juliana MM, et al Chemopreventive effects of the aromatase inhibitor vorozole (R 83842) in the methylnitrosourea-induced mammary cancer model. Carcinogenesis. 1998;19(8):1345–51. . 9744527 10.1093/carcin/19.8.1345

[3] Gottardis MM, Jordan VC. Antitumor actions of keoxifene and tamoxifen in the N-nitrosomethylurea-induced rat mammary carcinoma model. Cancer Res. 1987;47(15):4020–4. . 3607747

[4] Isaksson IM, Theodorsson A, Theodorsson E, Strom JO. Methods for 17beta-oestradiol administration to rats. Scand J Clin Lab Invest. 2011;71(7):583–92. 10.3109/00365513.2011.596944 . 21834617

[5] Rajkumar L, Guzman RC, Yang J, Thordarson G, Talamantes F, Nandi S. Short-term exposure to pregnancy levels of estrogen prevents mammary carcinogenesis. Proc Natl Acad Sci U S A. 2001;98(20):11755–9. 10.1073/pnas.201393798 . 11573010 PMC58802

[6] Kopper NW, Gudeman J, Thompson DJ. Transdermal hormone therapy in postmenopausal women: a review of metabolic effects and drug delivery technologies. Drug Des Devel Ther. 2009;2:193–202. . 19920906 10.2147/dddt.s4146 PMC2761184

[7] Stevenson JC. Type and route of estrogen administration. Climacteric. 2009;12 Suppl 1:86–90. . 19811249 10.1080/13697130903007389

[8] Blank EW, Wong PY, Lakshmanaswamy R, Guzman R, Nandi S. Both ovarian hormones estrogen and progesterone are necessary for hormonal mammary carcinogenesis in ovariectomized ACI rats. Proc Natl Acad Sci U S A. 2008;105(9):3527–32. 10.1073/pnas.0710535105 . 18299580 PMC2265202

[9] Ohi Y, Yoshida H. Influence of estrogen and progesterone on the induction of mammary carcinomas by 7,12-dimethylbenz(a)anthracene in ovariectomized rats. Virchows Arch B Cell Pathol Incl Mol Pathol. 1992;62(6):365–70. . 1360723 10.1007/BF02899705

[10] Bigsby RM. Synergistic tumor promoter effects of estrone and progesterone in methylnitrosourea-induced rat mammary cancer. Cancer Lett. 2002;179(2):113–9. . 11888665 10.1016/s0304-3835(02)00032-0

[11] Theodorsson A, Hilke S, Rugarn O, Linghammar D, Theodorsson E. Serum concentrations of 17beta-estradiol in ovariectomized rats during two times six weeks crossover treatment by daily injections in comparison with slow-release pellets. Scand J Clin Lab Invest. 2005;65(8):699–705. 10.1080/00365510500375206 . 16319044

[12] Jagger CJ, Chow JW, Chambers TJ. Estrogen suppresses activation but enhances formation phase of osteogenic response to mechanical stimulation in rat bone. J Clin Invest. 1996;98(10):2351–7. 10.1172/JCI119047 . 8941653 PMC507686

[13] Schindelin J, Arganda-Carreras I, Frise E, Kaynig V, Longair M, Pietzsch T, et al Fiji: an open-source platform for biological-image analysis. Nat Methods. 2012;9(7):676–82. 10.1038/nmeth.2019 . 22743772 PMC3855844

[14] Polanco TA, Crismale-Gann C, Reuhl KR, Sarkar DK, Cohick WS. Fetal alcohol exposure increases mammary tumor susceptibility and alters tumor phenotype in rats. Alcohol Clin Exp Res. 2010;34(11):1879–87. 10.1111/j.1530-0277.2010.01276.x . 20662802 PMC4634124

[15] Arqués O, Chicote I, Tenbaum S, Puig I, G. Palmer H. Standardized Relative Quantification of Immunofluorescence Tissue Staining. 2012. 10.1038/protex.2012.008

[16] Bartoshuk LM, Rifkin B, Marks LE, Hooper JE. Bitterness of Kcl and Benzoate—Related to Genetic Status for Sensitivity to Ptc/Prop. Chemical Senses. 1988;13(4):517–28. 10.1093/chemse/13.4.517. WOS:A1988R173900002.

[17] Gleason CE, Carlsson CM, Johnson S, Atwood C, Asthana S. Clinical pharmacology and differential cognitive efficacy of estrogen preparations. Ann N Y Acad Sci. 2005;1052:93–115. 10.1196/annals.1347.007 . 16024754

[18] Kupfer D. Critical evaluation of methods for detection and assessment of estrogenic compounds in mammals: strengths and limitations for application to risk assessment. Reprod Toxicol. 1987;1(2):147–53. . 2980373 10.1016/0890-6238(87)90010-4

[19] O'Connell MB. Pharmacokinetic and pharmacologic variation between different estrogen products. J Clin Pharmacol. 1995;35(9 Suppl):18S–24S. . 8530713 10.1002/j.1552-4604.1995.tb04143.x

[20] Longcope C, Gorbach S, Goldin B, Woods M, Dwyer J, Warram J. The metabolism of estradiol; oral compared to intravenous administration. J Steroid Biochem. 1985;23(6A):1065–70. . 4094413 10.1016/0022-4731(85)90068-8

[21] Strom JO, Theodorsson E, Theodorsson A. Order of magnitude differences between methods for maintaining physiological 17beta-oestradiol concentrations in ovariectomized rats. Scand J Clin Lab Invest. 2008;68(8):814–22. 10.1080/00365510802409703 . 18821130

[22] Haisenleder DJ, Schoenfelder AH, Marcinko ES, Geddis LM, Marshall JC. Estimation of estradiol in mouse serum samples: evaluation of commercial estradiol immunoassays. Endocrinology. 2011;152(11):4443–7. 10.1210/en.2011-1501 . 21933867 PMC3198998

[23] Witte MM, Resuehr D, Chandler AR, Mehle AK, Overton JM. Female mice and rats exhibit species-specific metabolic and behavioral responses to ovariectomy. Gen Comp Endocrinol. 2010;166(3):520–8. 10.1016/j.ygcen.2010.01.006 . 20067798 PMC2856744

[24] Chan M, Chow C, Hamson DK, Lieblich SE, Galea LA. Effects of chronic oestradiol, progesterone and medroxyprogesterone acetate on hippocampal neurogenesis and adrenal mass in adult female rats. J Neuroendocrinol. 2014;26(6):386–99. 10.1111/jne.12159 . 24750490

[25] Schwartz SM, Wade GN. Effects of estradiol and progesterone on food intake, body weight, and carcass adiposity in weanling rats. Am J Physiol. 1981;240(5):E499–503. . 7235006 10.1152/ajpendo.1981.240.5.E499

[26] Wade GN. Some effects of ovarian hormones on food intake and body weight in female rats. J Comp Physiol Psychol. 1975;88(1):183–93. . 1120795 10.1037/h0076186

[27] Wade GN. Gonadal hormones and behavioral regulation of body weight. Physiol Behav. 1972;8(3):523–34. . 4556652 10.1016/0031-9384(72)90340-x

[28] Newbold RR, Jefferson WN, Padilla-Banks E, Walker VR, Pena DS, Developmental Endocrinology Studies G. Cell response endpoints enhance sensitivity of the immature mouse uterotropic assay. Reprod Toxicol. 2001;15(3):245–52. . 11390168 10.1016/s0890-6238(01)00130-7

[29] Verdeal K, Rose DP, Erturk E, Harberg J. Induction of mammary tumors, estrous cycle abnormalities and endometrial hyperplasia in rats exposed to different doses of N-nitrosomethylurea. Eur J Cancer Clin Oncol. 1982;18(11):1171–80. . 6891656 10.1016/0277-5379(82)90099-2

[30] Liu J, Lin H, Huang Y, Liu Y, Wang B, Su F. Cognitive effects of long-term dydrogesterone treatment used alone or with estrogen on rat menopausal-models of different ages. Neuroscience. 2015 10.1016/j.neuroscience.2015.01.042 . 25637796

[31] Brisken C, Hess K, Jeitziner R. Progesterone and Overlooked Endocrine Pathways in Breast Cancer Pathogenesis. Endocrinology. 2015;156(10):3442–50. 10.1210/en.2015-1392 . 26241069 PMC4588833

[32] Jordan VC. Effect of tamoxifen (ICI 46,474) on initiation and growth of DMBA-induced rat mammary carcinomata. Eur J Cancer. 1976;12(6):419–24. . 821733 10.1016/0014-2964(76)90030-x

[33] Brisken C. Progesterone signalling in breast cancer: a neglected hormone coming into the limelight. Nat Rev Cancer. 2013;13(6):385–96. 10.1038/nrc3518 . 23702927

[34] Diep CH, Daniel AR, Mauro LJ, Knutson TP, Lange CA. Progesterone action in breast, uterine, and ovarian cancers. J Mol Endocrinol. 2015;54(2):R31–53. 10.1530/JME-14-0252 . 25587053 PMC4336822

[35] Mohammed H, Russell IA, Stark R, Rueda OM, Hickey TE, Tarulli GA, et al Progesterone receptor modulates ERalpha action in breast cancer. Nature. 2015;523(7560):313–7. 10.1038/nature14583 . 26153859 PMC4650274

[36] Beral V, Million Women Study C. Breast cancer and hormone-replacement therapy in the Million Women Study. Lancet. 2003;362(9382):419–27. . 12927427 10.1016/s0140-6736(03)14065-2

[37] Rossouw JE, Anderson GL, Prentice RL, LaCroix AZ, Kooperberg C, Stefanick ML, et al Risks and benefits of estrogen plus progestin in healthy postmenopausal women: principal results From the Women's Health Initiative randomized controlled trial. JAMA. 2002;288(3):321–33. . 12117397 10.1001/jama.288.3.321

[38] Fournier A, Berrino F, Clavel-Chapelon F. Unequal risks for breast cancer associated with different hormone replacement therapies: results from the E3N cohort study. Breast Cancer Res Treat. 2008;107(1):103–11. 10.1007/s10549-007-9523-x . 17333341 PMC2211383

[39] Mirkin S, Amadio JM, Bernick BA, Pickar JH, Archer DF. 17beta-Estradiol and natural progesterone for menopausal hormone therapy: REPLENISH phase 3 study design of a combination capsule and evidence review. Maturitas. 2015;81(1):28–35. 10.1016/j.maturitas.2015.02.266 . 25835751

[40] Grubbs CJ, Farnell DR, Hill DL, McDonough KC. Chemoprevention of N-nitroso-N-methylurea-induced mammary cancers by pretreatment with 17 beta-estradiol and progesterone. J Natl Cancer Inst. 1985;74(4):927–31. . 3857386

[41] Swanson SM, Christov K. Estradiol and progesterone can prevent rat mammary cancer when administered concomitantly with carcinogen but do not modify surviving tumor histology, estrogen receptor alpha status or Ha-ras mutation frequency. Anticancer Res. 2003;23(4):3207–13. . 12931682

[42] Kerdelhue B, Jolette J. The influence of the route of administration of 17beta-estradiol, intravenous (pulsed) versus oral, upon DMBA-induced mammary tumour development in ovariectomised rats. Breast Cancer Res Treat. 2002;73(1):13–22. . 12083627 10.1023/a:1015239128480

[43] Aupperlee MD, Haslam SZ. Differential hormonal regulation and function of progesterone receptor isoforms in normal adult mouse mammary gland. Endocrinology. 2007;148(5):2290–300. 17317767 10.1210/en.2006-1721

[44] Clarke RB, Howell A, Potten CS, Anderson E. Dissociation between steroid receptor expression and cell proliferation in the human breast. Cancer Res. 1997;57(22):4987–91. . 9371488

[45] Russo J, Ao X, Grill C, Russo IH. Pattern of distribution of cells positive for estrogen receptor alpha and progesterone receptor in relation to proliferating cells in the mammary gland. Breast Cancer Res Treat. 1999;53(3):217–27. . 10369068 10.1023/a:1006186719322

